# Improving compliance to colorectal cancer screening using blood and stool based tests in patients refusing screening colonoscopy in Germany

**DOI:** 10.1186/1471-230X-14-183

**Published:** 2014-10-17

**Authors:** Andreas Adler, Sebastian Geiger, Anne Keil, Harald Bias, Philipp Schatz, Theo deVos, Jens Dhein, Mathias Zimmermann, Rudolf Tauber, Bertram Wiedenmann

**Affiliations:** Central Interdisciplinary Endoscopy Unit, Department for Internal Medicine with focus on Hepatology, Gastroenterology and Metabolic Diseases, University Hospitals Berlin, Charité-Virchow-Klinikum, Berlin, Germany; Institute for Occupational Medicine, Charité-University Hospitals Berlin, Berlin, Germany; Berlin, Germany; Epigenomics Inc., Seattle, WA USA; Abbott Molecular, Wiesbaden, Germany; Institute for Laboratory Medicine, Clinical Chemistry and Pathobiochemistry, Charité – Universitätsmedizin Berlin and Labor Berlin, Charité Vivantes GmbH, Berlin, Germany

**Keywords:** Colorectal cancer, Screening, Non-invasive test, Blood test, Septin9, Colonoscopy, FIT

## Abstract

**Background:**

Despite strong recommendations for colorectal cancer (CRC) screening, participation rates are low. Understanding factors that affect screening choices is essential to developing future screening strategies. Therefore, this study assessed patient willingness to use non-invasive stool or blood based screening tests after refusing colonoscopy.

**Methods:**

Participants were recruited during regular consultations. Demographic, health, psychological and socioeconomic factors were recorded. All subjects were advised to undergo screening by colonoscopy. Subjects who refused colonoscopy were offered a choice of non-invasive tests. Subjects who selected stool testing received a collection kit and instructions; subjects who selected plasma testing had a blood draw during the office visit. Stool samples were tested with the Hb/Hp Complex Elisa test, and blood samples were tested with the Epi proColon® 2.0 test. Patients who were positive for either were advised to have a diagnostic colonoscopy.

**Results:**

63 of 172 subjects were compliant to screening colonoscopy (37%). 106 of the 109 subjects who refused colonoscopy accepted an alternative non-invasive method (97%). 90 selected the Septin9 blood test (83%), 16 selected a stool test (15%) and 3 refused any test (3%). Reasons for blood test preference included convenience of an office draw, overall convenience and less time consuming procedure.

**Conclusions:**

97% of subjects refusing colonoscopy accepted a non-invasive screening test of which 83% chose the Septin9 blood test. The observation that participation can be increased by offering non-invasive tests, and that a blood test is the preferred option should be validated in a prospective trial in the screening setting.

**Electronic supplementary material:**

The online version of this article (doi:10.1186/1471-230X-14-183) contains supplementary material, which is available to authorized users.

## Background

Colorectal cancer (CRC) has been estimated to afflict 1.36 million people worldwide, accounting for nearly 10% of cancers [1] and is the second most common cause of death due to cancer in Europe [2]. It is well established that the five-year survival rate for CRC, which is greater than 90% for early localized cancer, drops to less than 5% for late stage metastatic disease. A number of CRC screening methods aimed at early detection have been developed, and there is a substantial body of evidence supporting the benefits of CRC screening [3–5]. Paradoxically, despite the clear and long standing evidence that CRC screening reduces mortality and may reduce cancer incidence, participation rates in screening programs remains too low, at an estimated 65% in the US [6] and ranging from 1.9% to 54% across Europe [7].

In Germany, screening by annual guaiac fecal occult blood tests (gFOBT) has been recommended since the mid 1970’s, and screening by colonoscopy was introduced as a covered option in 2002 [8, 9]. While screening is encouraged, organized nationwide screening activities are limited. Both methods are available without additional cost as part of health care coverage. Estimates of test usage in Germany indicate that for use of FOBT within the past year, only 14% of men and 22% of women were compliant, and that colonoscopy use within 10 years was 23% for men and 26% for women [8] though in most instances the colonoscopy was diagnostic rather than for screening. In Germany, nationwide data on screening colonoscopy, including adenoma detection, cecal intubation and complication rates amongst others are tracked through a central registry [10]. In the city of Berlin, the quality and performance of screening colonoscopy has been tracked through the Berlin colonoscopy projects - BECOP 1&3 [9, 11].

Given the low participation in CRC screening programs despite the clear medical benefit, it is important to understand the barriers to screening to develop successful alternative approaches. Numerous studies report behavioral as well as structural barriers that limit screening participation. These include factors specific to the tests themselves, such as embarrassment, fear of the procedure, or inconvenience, as well as broader factors such as lack of access to care, limited knowledge of screening and a lack of physician recommendation [reviewed in 12]. While these findings are clearly influenced by the country or health system of the participants, many factors (e.g. fear) are consistently reported in different settings [12]. To overcome these barriers, considerable effort has gone in to developing educational and outreach programs to improve screening rates. One aspect of this has been the demonstration that offering a choice in tests has a positive impact on participation in screening programs [13].

As indicated above, surveys, focus groups and patient interviews focused on understanding the resistance to screening, demonstrate that the screening modalities themselves (fecal sampling, bowel prep, colonoscopy etc.) present significant hurdles to patients [12]. The blood based test for CRC screening provides an alternative screening method based on the detection of methylated Septin9 DNA in patient plasma, and may overcome these barriers [14]. The test uses a standard EDTA plasma sample collected at the physician’s office or diagnostic laboratory.

The performance characteristics of the Epi proColon 2.0 CE blood test have been reported to be in the range of 70% sensitivity and 90% specificity in a number of studies [14–17]. The test has no dietary or time restrictions. The objective of the current study was to determine the impact of offering a new blood based test on the participation rate for CRC screening in Berlin, Germany rather than on characterizing performance. In addition, the study aimed to determine the relationships between demographic variables and test choice, and to survey the reasons for choosing non-invasive tests.

## Methods

**Figure 1:**
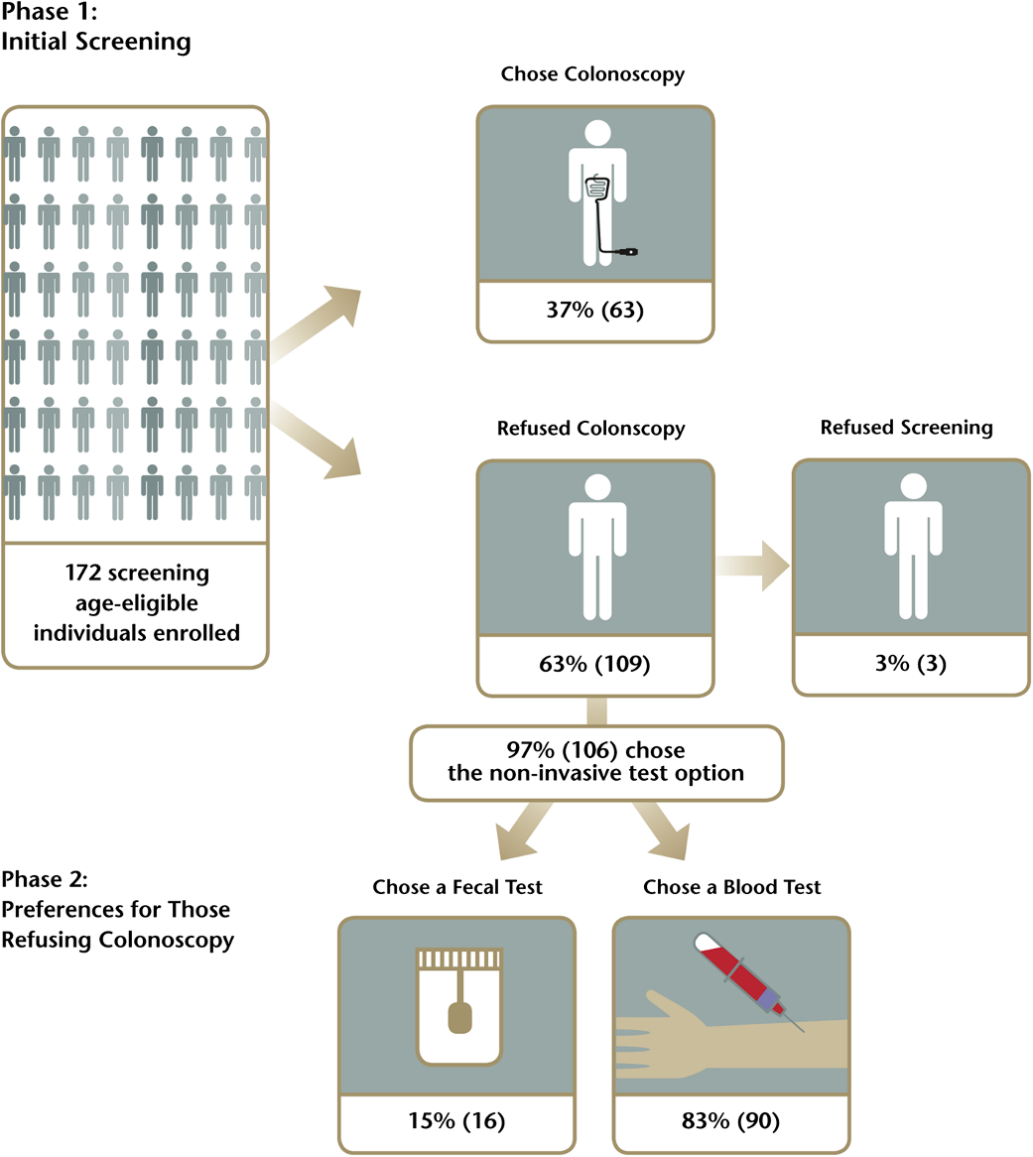
**Flow chart outline of the study design.** The number of subjects enrolled, the number refusing colonoscopy, and the number choosing a blood test, stool test, or refusing any test are indicated.

The study protocol was reviewed and approved by the Institutional Review Board of the Charite hospital. Subjects were recruited at 15 sites in Berlin (Germany) and surrounding areas, all of which were managed under the Charite hospital review board. All subjects provided written informed consent. Inclusion criteria were: age 50 to 75; Patient showing no symptoms that may indicate a tumor of the colon; Patient has no known inflammatory bowel disease; Patient has no known familial predisposition for colorectal cancer; Patient has no known strong family history or genetic predisposition to colorectal cancer; Informed Consent provided. The study design is summarized in Figure 1.

### Study subjects

Subjects were recruited during regular consultations with primary care physicians or company doctors as outlined in the Additional file 1. Physicians followed a standardized recommendation script, first offering screening by colonoscopy, and for patients who refused, offered the option of non-invasive testing with the stool based immunochemical fecal occult blood test (FIT) or the Septin9 blood test. Although the protocol stated that all patients fitting the inclusion criteria be asked to participate, the total number of potential patients who could be invited to be screened was not recorded. 174 CRC screening eligible subjects were enrolled and provided demographic, health, psychological and socioeconomic data by a questionnaire (Tables 1, 2 and 3). Two subjects who were younger than 50 were removed from the analysis. Although the recommended age for screening by colonoscopy is 55 in Germany, screening by FIT/FOBT is promoted for patients aged 50+ and therefore all subjects age 50+ were included in the analysis. All subjects were advised to undergo screening by colonoscopy and the screening decision was recorded. Subjects who accepted colonoscopy were not included in the remainder of the study.

**Table 1:** **Demographic information of all subjects enrolled in the study**

		Total	Refused colonoscopy		
			N	%	95% CI*
Enrolled		172	109	63.4	
Gender					
	Female	104	69	66.3	56.8–74.7
	Male	67	40	59.7	47.7–70.6
Age					
	50–60	70	44	62.9	51.1–73.2
	60–69	65	45	69.2	57.2–79.1
	70+	33	17	51.5	35.2–67.5
Ethnicity					
	German	137	89	65.0	56.7–72.4
	Not German	35	20	57.1	40.9–72.0
Education					
	None / Basic	69	43	62.3	50.5–72.8
	A level / University	82	48	58.5	47.7–68.6
Proffession/Work					
	Employed	88	55	62.5	52.1–71.9
	Retired	71	43	60.6	48.9–71.1
	Unemployed	7	6	85.7	48.7–99.3
Monthly income					
	≤1000 €	36	29	80.6	65.0–90.2
	1001–2000 €	75	43	57.3	46.1–67.9
	>2000 €	30	19	63.3	45.5–78.1
	No Data	31	18	58.1	40.8–73.6
Health insurance					
	State	158	101	63.9	56.2–71.0
	Private	12	8	66.7	39.1–86.2
	No data	2	0	0	

*Confidence Interval.

**Table 2:** **Self reported health information for all subjects enrolled in the study**

		Total	Refused colonoscopy		
			N	%	95% CI*
Health status					
	Good	130	82	63.1	54.5–70.9
	Poor	41	26	63.4	48.1–76.4
Self evaluated – Risk of CRC					
	Small	148	97	65.5	57.6–72.7
	Large	23	11	47.8	29.2–67.0
Frequency of regular exercise**					
	0–1	81	39	48.1	37.6–58.9
	1+	91	70	76.9	67.3–84.4
Diet					
	Calorie Rich, Sweets, Animal Fats and Red Meat	18	8	44.4	24.6–66.3
	Fresh Fruit, Vegetables, White Meat and Fish	57	35	61.4	48.4–72.9
	Varied – a bit of everything	97	66	68.0	58.2–76.5
Alcohol consumption					
	Up to 2–3 per week	155	98	63.2	55.4–70.4
	Daily	14	8	57.1	32.6–78.6
Smoking					
	Non Smoking	134	85	63.4	55.0–71.1
	Smoking	38	24	63.2	47.3–76.6
Number of physician visits in last year					
	<15	44	30	68.2	53.4–80.0
	15+	123	75	61.0	52.1–69.1

*Confidence Interval.

**Significant based on χ^2^ test.

**Table 3:** **Patient knowledge related to cancer, colorectal cancer and gastro-intestinal health for all subjects enrolled in the study**

		Total number of subjects	Refused colonoscopy		
			N	%	CI
Prior experience with gastro-intestinal inflammation or polyps					
	Yes	22	10	45.5	26.9–65.3
	No	150	99	66.0	58.1–73.1
Prior Experience with any form of Cancer (excluding colorectal cancer)					
	Yes	13	6	46.2	23.2–70.9
	No	158	102	64.6	56.8–71.6
	No Data	1	1	100	5.1–100.0
Any family members with polyps					
	Yes	17	8	47.1	26.2–69.0
	No	120	75	62.5	53.6–70.6
	No Data	35	26	74.2	57.9–85.8
Any family members diagnosed with cancer					
	Yes	79	52	65.8	54.8–75.3
	No	91	55	60.4	50.2–69.9
	No Data	1	1	100.0	5.1–100.0

Subjects who refused screening colonoscopy were offered the option of either a FIT test (Hb / Hp–Complex ELISA, MDI Laboratorien GmbH, Berlin Germany) or an Epi proColon blood test (Epigenomics AG, Berlin, Germany). Physicians provided approved descriptive materials for each test. These subjects also filled out an additional questionnaire focused on determining the basis for their screening decisions (Additional file 1: Table S1–S11).

### Laboratory testing

Subjects who selected a stool test were provided the Hb/Hp–Complex ELISA stool collection kit, instructions for sample collection, and were requested to provide the fecal sample to the testing lab. Subjects who selected the plasma test provided a blood sample as part of the physician visit. For both methods, tests were performed following the manufacturers instruction at a qualified laboratory and the test results were recorded. Individuals who were positive for either test were recommended to have a diagnostic colonoscopy.

### Statistical analysis

Answers from the questionnaires were reported as simple numbers and percentages. The significance of demographic, health, psychological and medical variables between colonoscopy acceptors and refusers were analysed by the Chi squared test, (p < 0.05). To correct for age, logisitic regression models were used with acceptor/refuser as response and age as an additional variable.

## Results

Of the 174 subjects enrolled, 2 were under the age of 50 and were excluded. Sixty three (36.6%) opted for screening colonoscopy and 109 (63.4%) refused (Figure 1). Demographic data for the 172 included subjects is summarized in Table 1 and the number refusing colonoscopy is indicated.

Self reported health information is reported in Table 2, and knowledge of cancer, colorectal cancer and general gastrointestinal health is outlined in Table 3, and the numbers refusing colonoscopy are indicated. For a number of parameters, a more detailed breakdown is reported in the Additional file 1: Table S1. Subject variables were analyzed using the Chi squared test to determine differences between colonoscopy acceptors and refusers. Significant differences were observed for the amount of weekly exercise (p ≤ 0.001) where those who excercised more frequently were also more likely to opt for a non-invasive test than a colonoscopy (Table 4). In the analysis with additional subgroups, subjects perceived CRC risk (p ≤ 0.01) was also significant, while factors such as monthly income and education level were close to significant (Table 4). While these variables were significant, no trend was observed for these variables. When further analyzed using logistic regression models with age as an additional variable, only level of exercise remained significant (p < 0.01). The remaining variables did not differ significantly at the p ≤ 0.05 level. General awareness of screening methods and prior use of tests is presented in Table 5, which illustrates that 95% of participants had an awareness of colorectal cancer screening tests.

**Table 4:** **Analysis of accepting/refusing colonoscopy for demographic parameters with detailed and collapsed categories (χ** ^**2**^ **– Test) and p-values of likelihood ratio test (LRT) for collapsed parameters including age**

Parameter	P value detailed parameters (χ ^2^ – Test)	P value collapsed* parameters (χ ^2^ – Test)	Likelihood ratio test – p value
Gender	0.47		0.60
Age Group	0.23		1.00
Ethnicity	0.37	0.51	0.29
Education	0.07	0.76	0.08
Profession/Work	0.60	0.42	0.34
Monthly Income	0.06		0.03
Health Insurance	1.00		0.82
Health Status	0.59	1.00	0.98
Self evaluated risk of CRC	0.01	0.16	0.005
Frequency of regular exercise	<0.001	<0.001	0.0004
Diet	0.15		0.09
Alcohol Consumption	0.48	0.87	0.47
Smoking	0.34	1.00	0.40
Number of physician visits in last year	0.19		0.27
Prior experience inflammation or polyps	0.10		0.08
Prior Experience - Cancer (excluding CRC)	0.31		0.31
Any family members with polyps	0.34		0.18
Any family members diagnosed with cancer	0.57		0.76

*For some demographic variables, classes were combined to increase the number of subjects, and were analyzed (compare Table 1 and Additional file 1: Table S1).

**Table 5:** **Prior knowledge of CRC screening tests**

Awareness of CRC Screening Tests	N (%)	CI
Fecal occult blood test	157 (91%)	86–95%
Colonoscopy	162 (94%)	90–97%
Sigmoidoscopy	22 (13%)	9–19%
Barium contrast enema	28 (16%)	12–23%
Septin9 blood test	36 (21%)	16–28%

The subjects who refused colonoscopy were offered non-invasive screening. Of the 109 subjects who rejected colonoscopy, 90 (82.6%) opted for the blood test, 16 (14.7%) opted for the Hb/Hp–Complex ELISA FOBT test, and 3 (2.8%) refused both options (Figure 1). These subjects also answered questions related to their screening decision. As indicated in the Additional file 1: Table S2, the top three reasons for rejecting colonoscopy were being uncomfortable with the bowel preparation for colonoscopy (54%), fear of colorectal cancer itself (44%) and fear that colonoscopy would be painful (32%). These results were corroborated in a follow-up question asking what would convince subjects to be screened by colonoscopy where 38% indicated an improved bowel preparation, 29% indicated cancer prevention by polypectomy and 24% indicated that overcoming fears would change their minds (Additional file 1: Tables S3). In addition, when asked why they chose one of the screening tests, 78% and 81% of subjects who had a blood and stool test respectively, indicated ease of getting the test (Additional file 1: Tables S4, S5, S7, S8). For those choosing the blood test, primary reasons for not choosing the stool test related to being uncomfortable with specimen handling (Additional file 1: Table S6). For those choosing the stool test, the primary reason related to having used a stool test in the past (Additional file 1: Table S9). As only 3 subjects rejected any form of testing, limited survey data is available (Additional file 1: Table S10 through S12).

Finally, though not the focus of this study, the two subjects who were positive for the Septin9 test and the two who were positive for FIT went on to colonoscopy.

## Discussion

In this observational study, we report the impact of providing a choice of non-invasive screening tests on participation in colorectal cancer screening in Berlin, Germany and surrounding areas. We also report the results of surveys of participants addressing their perspectives on the different screening test options. In this study, 36.6% of participants chose to have a screening colonoscopy. Among the 63.4% who refused colonoscopy, 82.6% selected the Septin9 blood test, 14.7% selected the stool test and 2.8% refused any test. Thus, when all methods were considered, screening levels reached 98% (169/172 subjects).

Study recruitment was undertaken in Occupational Health and Primary Care settings, and therefore the following comparison of some of the key demographic data (Table 1) was made with census data from the German population [18]. The study enrolled a higher proportion of women (60.8%) which may be explained in part by the elevated ratio of women in the eligible age population (~53%) [18]. The age distribution was representative, with lower numbers enrolled in the 70+ age group compared to the total population (20% in the study vs 34% in the population) which reflects enrollment in the Occupational Health setting. Comparison of the migrant status of enrolled subjects was similar to that for the Berlin region, with 20.3% not German in the study, compared with 24% with a migrant background in the population [18]. The observed un-employment rate in the population was as expected (4.2% in the study, 4.6% in the age matched population) as were the rates of employed and retired subjects. Finally, the distribution of education level in the current study was higher on average than in the general population (56% no or lower education in the population compared with 40.4% in the study) though using the same metric for the Berlin region, the population estimate was 38% for no or lower education, which compares well with the 40.4% observed in the study [18]. While differences from the overall German population are not unexpected, given the sample size and the regional location of the study, the different demographic strata are represented. They are close to the observed levels for the Berlin region, thus allowing for extrapolation of the results to the region, and with caution, are also informative for the broader German population.

Given that enrollment was in a setting where CRC screening was promoted, this may account for the high degree of screening knowledge observed in the study. While the rate of acceptance of screening colonoscopy in this study (37%) was higher than the overall reported rate for the German population (~25%) [8], it remained well below that reported for the US. It is unclear why the rate of screening colonoscopy is higher than usually observed in Germany, though it may be attributed in part to participation in a study. As the study focused on the screening population, subjects were asymptomatic and representative of the broader population. It may however, also represent an overestimate, since the number refusing enrollment was not recorded.

There are only a few reports on barriers to acceptance of colonoscopy in the German screening population. In a 2009 report from the Leipzig area, the primary reasons for not being screened were a lack of awareness or recommendation for screening [19]. In a detailed survey from the Munich area, fear of the bowel preparation, lack of a physician recommendation and a lower interest in screening were associated with avoiding colonoscopy [20]. Interestingly, in that study, the demographics associated with having a colonoscopy were: lower education status, unemployment or retired, or having a primary care physician [20].

As shown in the detailed analysis in the supplement, a similar trend was observed for participants who completed Grade School compared with those completing A levels, though this was reversed for those who completed university. When these categories were aggregated, the difference was not significant. These trends differ from the US, where the lowest screening rates correlate with low socioeconomic status indicators such as income, education level and lack of work [12], and this illustrates the importance of developing an understanding of the issues at a local level. It is also interesting to note that we did not observe a correlation between refusal of colonoscopy and other health factors such as diet, alcohol consumption or smoking status.

In the current study, the primary reasons given for not having a colonoscopy were associated with fear, discomfort or concern about the bowel preparation or the colonoscopy procedure. This outcome suggests that better education about the procedure is a possible course for increasing screening by colonoscopy. In addition the primary reasons patients provided for selecting non-invasive tests related to convenience of use, and the selection of a blood test over a stool test was based on a preference not to handle stool samples. Thus, it appears that the preference for the blood test is not necessarily related to the performance of the test, but rather the convenience it offers with blood collection available at the physician’s office.

We observed that 97% of the participants who refused a colonoscopy were willing to accept a non-invasive test, despite these tests having lower performance outcomes. This aligns with the observation that educating patients with the evidence for the benefits of colonoscopy versus other screening methods had no impact on their attitude to CRC screening or their ultimate test choice [21]. Thus, understanding the patient’s motivation for screening is crucial to developing successful programs. This is underscored in a recent trial, where offering screening alternatives increased overall participation in a screening program [13]. It is further illustrated by the experience at Kaiser-Permanente in California, where, following failed efforts to implement screening by endoscopy, screening participation has consistently increased with the re-introduction of a non-invasive FIT test [22], despite the test having lower sensitivity.

It is clear from the results of the current study that offering a blood test as part of the screening menu further improves participation, as approximately 80 percent of subjects opted for a blood test. It is important to note that in the present study, the tests were provided at no cost to participants. As the blood test is not currently covered under national health care, the impact of cost to patient needs further analysis. Based on the survey data, key factors in the decision to be screened with the blood test were trust of blood tests, being comfortable with giving blood and the convenience of a blood draw compared with providing a stool sample. It is also interesting to note that providing test choice can improve screening participation, similar to what was observed by Inadomi et.al. [13] in a study in California, as well as in a discrete choice study in the Netherlands [23].

While it is clear that the ease and convenience of a blood test can improve screening participation, there are many additional factors that will determine the impact of a new screening test. In addition to performance characteristics, these include guideline recommendations, health economic considerations, and cost to patient amongst others. Despite this complex landscape, which includes significant differences in philosophy and approach by region and country, the data in this study support the idea that test convenience is an important consideration in the success of CRC screening programs.

With the limited sample size, and the observation of only a small number of patients who were positive for either non-invasive test, we did not perform statistical analysis on the test results, or whether subjects with a positive test result went on to colonoscopy. Anecdotally, the two subjects who were positive for the Septin9 test and the two who were positive for FIT went on to colonoscopy. Clearly, compliance with follow-up diagnostic colonoscopy is a critical aspect in the success of a screening program. In all four subjects, adenomas were removed completely during colonoscopy. One patient of the Septin9 group showed high grade intraepithelial neoplasia and the others had low grade intraepithelial neoplasias.

### Limitations of the study

The study was subject to a number of limitations. 1) It was designed to enroll 100 subjects who refused colonoscopy. While this sample size was deemed sufficient to assess general preference for the two non-invasive screening modalities, it allows only limited observational analysis of subgroups. 2) By protocol, the study was designed to enroll all eligible subjects in each practice. However, the study did not include a mechanism to record the total number of subjects asked to participate or the number who refused to participate. In this respect, the results cannot be presented in the context of ‘intention to screen’. As a result, there may be bias in the study, resulting in the higher than expected estimation of participation rates. 3) The study was performed in a limited geographical setting (Berlin, Germany) and therefore, extrapolation to a broader national or broader European context should be done with caution.

## Conclusions

The results of this study support the contention that providing non-invasive screening choices can augment participation in programs for colorectal cancer screening. The success of a screening program depends on having tests with acceptable performance, but also on the willingness of the target population to participate. This study demonstrates that offering non-invasive test options significantly increases compliance to colorectal cancer screening. Furthermore the addition of a convenient blood test that can be provided in the physician’s office, has the potential to improve screening participation.

Future studies will focus on characterization of complete screening programs, from the invitation to screening through to the completion of colonoscopy for patients with positive tests.

## Electronic supplementary material

Additional file 1: **Preference Data Supplement.** (DOCX 29 KB)
