# Supplementary material for: Improving compliance to colorectal cancer screening using blood and stool based tests in patients refusing screening colonoscopy in Germany
Source: BMC Gastroenterol. 2014 Oct 17;14:183. doi: 10.1186/1471-230X-14-183 (PMC4287474; doi:10.1186/1471-230X-14-183)
Supplement: Supplementary file 1 — Additional file 1: Preference Data Supplement. (DOCX 29 KB) [file 12876_2014_1204_MOESM1_ESM.docx]

Additional file 1:

Patient Recruitment :

Patients meeting the inclusion criteria were asked during a visit to the family doctor or company doctor if they wish to participate in a survey for colorectal cancer screening. After reconnaissance and successful consent, a questionnaire for socio-demographics, subjective health perception, risk factors and medical competence was presented to be filled out by the patient. Afterwards, the patient was informed in a standardized interview about colonoscopy for colorectal cancer screening.

Physicians had a 2 part interview guide at hand as guidance documents. The interview guides were created in accordance with the recommendations of the Bavarian Statutory Health Insurance. The first part described the individual personal risk for colorectal cancer (CRC) and the environmental risk factors for getting CRC. It ended with the description of colonoscopy as screening gold standard with its specificity and sensitivity. If the patient refused the colonoscopy, they were informed about non-invasive alternative screening methods with second part of the interview guide.

Patients who refused colonoscopy were requested to participate in a study to investigate new methods for colorectal cancer screening. These participants of the second study part were offered an immunological FOB test (FIT) (Hb / Hp Complex - test) and the blood-based Septin 9 test. Both tests were described as showing comparable sensitivity and specificity. Patients who opted to take the FIT were provided a test kit for home use, to be returned to the laboratory. Patients who opted to take the Septin9 test could give a blood test as part of the physician visit.

Table S1 – Expanded demographic parameters for all subjects enrolled in the study.

| Parameter | Total | Refused Colonoscopy | | |
| --- | --- | --- | --- | --- |
|  |  | N | % | 95% CI* |
| Enrolled | 172 | 109 | 63.4 |  |
| Age** |  |  |  |  |
| 50-60 | 70 | 44 | 62.8 | 51.1-73.2 |
| 61-70 | 65 | 45 | 69.2 | 57.2-79.1 |
| >70 | 33 | 17 | 51.5 | 35.2-67.5 |
| No Data | 4 | 3 | 75 |  |
| Ethnicity |  |  |  |  |
| German | 137 | 89 | 65.0 | 56.7-72.4 |
| Turkish | 5 | 4 | 80.0 | 37.6-99.0 |
| Russian | 3 | 2 | 66.7 | 20.8-98.3 |
| Other | 10 | 4 | 40.0 | 16.8-68.7 |
| No Data | 17 | 10 | 58.8 | 36.0-78.4 |
| Education |  |  |  |  |
| None | 3 | 2 | 66.6 | 20.8-98.3 |
| Grade School | 66 | 41 | 62.1 | 50.1-72.9 |
| A Levels | 23 | 17 | 73.9 | 53.5-87.5 |
| University | 59 | 31 | 52.5 | 40.0-64.7 |
| Other | 21 | 18 | 85.7 | 65.4-95.0 |
| Profession / Work |  |  |  |  |
| Employee | 69 | 42 | 60.9 | 49.1-71.5 |
| Management | 6 | 4 | 66.7 | 30.0-90.3 |
| Self Employed | 10 | 6 | 60.0 | 31.3-83.2 |
| Homemaker | 3 | 3 | 100.0 | 43.9-100.0 |
| Unemployed | 7 | 6 | 85.7 | 48.7-99.3 |
| Retired | 71 | 43 | 60.6 | 48.9-71.1 |
| Other | 6 | 5 | 83.3 | 43.6-99.1 |
| Health Status |  |  |  |  |
| Very Poor | 3 | 3 | 100 | 43.9-100.0 |
| Poor | 38 | 23 | 60.5 | 44.7-74.4 |
| Good | 118 | 74 | 62.7 | 53.7-70.9 |
| Very Good | 12 | 8 | 66.7 | 39.1-0.862 |
| No Data | 1 | 1 | 100 |  |
| Self Evaluated – Risk of CRC** |  |  |  |  |
| Very small | 24 | 20 | 83.3 | 64.1-93.3 |
| Small | 124 | 77 | 62.1 | 53.3-70.2 |
| Large | 17 | 6 | 35.3 | 17.3-58.7 |
| Very Large | 6 | 5 | 83.3 | 43.6-99.1 |
| No Data | 1 | 1 | 100 |  |
| Frequency of Regular Exercise** |  |  |  |  |
| None | 43 | 16 | 37.2 | 24.4-52.1 |
| 1x Weekly | 38 | 23 | 60.5 | 44.7-74.4 |
| 2-3 x Weekly | 52 | 43 | 82.7 | 70.3-90.6 |
| Daily | 39 | 27 | 69.2 | 53.6-81.4 |
| Alcohol Consumption |  |  |  |  |
| ≤ 1x per week | 109 | 72 | 66.1 | 56.8-74.3 |
| 2-3 x per week | 46 | 26 | 56.5 | 42.2-69.8 |
| Daily | 14 | 8 | 57.1 | 32.6-78.6 |
| No Data | 3 | 3 | 100 | 43.9-100.0 |
| Smoking |  |  |  |  |
| Do not smoke | 134 | 85 | 63.4 | 55.0-71.1 |
| ≤ 1 pack per day | 23 | 16 | 69.5 | 49.1-84.4 |
| ≤ 1 pack per week | 5 | 4 | 80 | 37.6-99.0 |
| ≥ 1 pack per day | 10 | 4 | 40 | 16.8-68.7 |
| Number of physician visits in last year |  |  |  |  |
| <5 | 84 | 56 | 66.7 | 56.1-75.8 |
| 5-15 | 59 | 35 | 59.3 | 46.6-70.9 |
| 15-25 | 14 | 7 | 50 | 26.8-73.2 |
| >25 | 5 | 2 | 40 | 11.8-76.9 |
| No Data | 10 | 9 | 90 |  |

* Confidence Interval

** Variables that were significantly associated with non-invasive testing (p<0.05)

Table S2. Why did you decide not to do a colonoscopy?

| Response | Blood n=90 | Stool n=16 | Refuse  N=3 | Total n=109 | Rank |
| --- | --- | --- | --- | --- | --- |
| I am not comfortable with the test or with the subject “colon cancer” | 39 | 7 | 2 | 48 | 2 |
| I am not comfortable with the preparation necessary for colonoscopy | 50 | 7 | 2 | 59 | 1 |
| I am at low risk for colon cancer | 14 | 4 | 1 | 19 | 6 |
| I am healthy and do not need to be screened | 7 | 1 | 0 | 8 | 10 |
| I have not thought about this topic | 8 | 0 | 1 | 9 | 9 |
| I don’t have time for the bowel preparation | 10 | 1 | 0 | 11 | 8 |
| I am scared of a cancer diagnosis | 13 | 1 | 1 | 15 | 7 |
| I believe that colonoscopy is painful | 30 | 4 | 1 | 35 | 3 |
| I believe that colonoscopy will cause physical stress | 20 | 2 | 0 | 22 | 4 |
| The idea of colonoscopy is stressful | 18 | 3 | 0 | 21 | 5 |
| My physician did not recommend colonoscopy | 1 | 0 | 0 | 1 | 13 |
| Friends advised against colonoscopy | 2 | 0 | 0 | 2 | 12 |
| I don’t know | 6 | 1 | 0 | 7 | 11 |

Table S3. What would make you change your mind and have a colonoscopy?

| Response | Blood  N=90 | Stool N=16 | Refuse N=3 | Total N=109 | Rank |
| --- | --- | --- | --- | --- | --- |
| Prevention of cancer by removing pre-cancer during colonoscopy | 27 | 4 | 1 | 32 | 2 |
| Have more time | 5 | 0 | 0 | 5 | 7 |
| Simplification of bowel preparation | 35 | 5 | 1 | 41 | 1 |
| Better overall health | 6 | 1 | 2 | 9 | 5 |
| Overcome my fears | 21 | 4 | 1 | 26 | 3 |
| Physician recommendation | 12 | 2 | 1 | 15 | 4 |
| Friends recommendation | 0 | 0 | 1 | 1 | 8 |
| Don’t know | 13 | 1 | 1 | 15 | 4 |
| I would never change my mind. | 4 | 2 | 0 | 6 | 6 |

Table S4 Why did you choose a blood test?*

| Answer | Subjects  N=90 |
| --- | --- |
| The ease / comfort of a blood test | 71 |
| The blood test was recommended to me | 43 |
| The convenience of giving blood at the doctor’s office | 67 |
| A positive blood test result does not necessarily mean that I have colon cancer | 12 |
| The blood draw was time saving | 43 |
| I don’t know | 0 |

*Subjects were given the list of possible answers and selected all applicable to them. The number represents the number of times an answer was selected, such that answers with the highest number were selected most frequently.

Table S5. What were the advantages of the blood test compared with colonoscopy?*

| Answer | Subjects N=90 |
| --- | --- |
| I do not feel comfortable with colonoscopy | 60 |
| I prefer a lab test compared with colonoscopy | 43 |
| The test is less painful | 45 |
| I do not have to go through the bowel preparation needed for colonoscopy | 45 |
| The blood test is less stressful for my health | 20 |
| I prefer the convenience of not having to come back for the procedure | 4 |
| I can discuss the result with my GP/company doctor | 19 |
| I trust in measured values than in colonoscopy | 8 |
| A blood test is less mentally stressful | 37 |
| I don’t know | 5 |

*Subjects were given the list of possible answers and selected all applicable to them. The number represents the number of times an answer was selected, such that answers with the highest number were selected most frequently.

Table S6. What are the advantages of the blood test compared to the stool test?*

| Answer | Subjects N=90 |
| --- | --- |
| I don’t feel comfortable with the submission of a stool sample | 23 |
| I don’t have to collect the sample myself. It is in the responsibility of the physician/laboratory | 25 |
| I don’t need to store stool samples in my refrigerator | 30 |
| I could complete my test in one visit | 10 |
| I did not have to restrict my diet or change my medication | 25 |
| A blood test is more accurate | 32 |
| I trust a blood test more than a stool test | 43 |
| My toilet is not suitable for taking a stool sample | 15 |
| I don’t know | 6 |

*Subjects were given the list of possible answers and selected all applicable to them. The number represents the number of times an answer was selected, such that answers with the highest number were selected most frequently.

Table S7. Why did you choose a stool test?*

| Answer | Subjects N=16 |
| --- | --- |
| The simplicity of the stool test | 13 |
| The stool test was recommended to me | 7 |
| A positive stool test does not necessarily mean that I have cancer | 1 |
| I don’t know | 2 |

*Subjects were given the list of possible answers and selected all applicable to them. The number represents the number of times an answer was selected, such that answers with the highest number were selected most frequently.

Table S8. What are the advantages of the stool test compared to the colonoscopy for you?*

| Answer | Subjects N=16 |
| --- | --- |
| I did not feel comfortable with the colonoscopy | 9 |
| I prefer a lab test compared to the colonoscopy | 4 |
| The test is less painful | 5 |
| I don’t have to go through the bowel preparation necessary for colonoscopy | 6 |
| The stool test is less stressful for my health | 3 |
| I don’t know any gastroenterologist | 0 |
| I can discuss the result with my GP/company doctor. | 3 |
| I trust in measure values than in colonoscopy | 0 |
| A stool test is psychologically less stressful | 7 |
| I don’t know | 1 |

*Subjects were given the list of possible answers and selected all applicable to them. The number represents the number of times an answer was selected, such that answers with the highest number were selected most frequently.

Table S9. What are the advantages of the stool test compared to the blood test?*

| Answer | Subjects N=16 |
| --- | --- |
| I feel uncomfortable with having a blood draw | 0 |
| I am scared of the blood draw | 0 |
| A stool test is more accurate | 5 |
| I trust a stool test more than a blood test | 5 |
| I am used to submitting stool tests for CRC screening | 9 |
| I don’t know | 4 |

*Subjects were given the list of possible answers and selected all applicable to them. The number represents the number of times an answer was selected, such that answers with the highest number were selected most frequently.

Table S10. You refused any testing. Which test would you take if you had to make a choice?

| Answer | Subjects N=3 |
| --- | --- |
| Blood Test | 2 |
| Stool Test | 1 |

Table S11. What would you need to change to convince you to take one of the non-invasive tests?

| Answer | Subjects N=3 |
| --- | --- |
| The test must be reimbursed by the social health insurance | 1 |
| The test should be recommended to me by my GP | 1 |
| I would need to know more about the test | 1 |
| The test should be less expensive | 0 |
| I don’t know | 1 |

Table S12. What would you need to change to convince you to take the other of the non-invasive tests?

| Answer | Subjects N=3 |
| --- | --- |
| The test must be reimbursed by the social health insurance | 1 |
| The test should be recommended to me by my GP | 1 |
| I would need to know more about the test | 0 |
| The test should be less expensive | 0 |
| I don’t know | 1 |
